# Characterization and Curing Kinetics of Epoxy/Silica Nano-Hybrids

**DOI:** 10.3390/ma8105357

**Published:** 2015-10-16

**Authors:** Cheng-Fu Yang, Li-Fen Wang, Song-Mao Wu, Chean-Cheng Su

**Affiliations:** 1 Department of Chemical and Materials Engineering, National University of Kaohsiung, No. 700, Kaohsiung University Rd., Nan-Tzu Dist., Kaohsiung 811, Taiwan; cfyang@nuk.edu.tw; 2 Department of Applied Chemistry and Materials Science, Fooyin University, 151 Jinxue Rd., Daliao Dist., Kaohsiung 831, Taiwan; sc112@fy.edu.tw; 3 Department of Electrical Engineering, National University of Kaohsiung, No. 700, Kaohsiung University Rd., Nan-Tzu Dist., Kaohsiung 811, Taiwan; sungmao@nuk.edu.tw

**Keywords:** sol-gel technique, nanocomposite, differential scanning calorimetry (DSC), autocatalytic mechanism

## Abstract

The sol-gel technique was used to prepare epoxy/silica nano-hybrids. The thermal characteristics, curing kinetics and structure of epoxy/silica nano-hybrids were studied using differential scanning calorimetry (DSC), ^29^Si nuclear magnetic resonance (NMR) and transmission electron microscopy (TEM). To improve the compatibility between the organic and inorganic phases, a coupling agent was used to modify the diglycidyl ether of bisphenol A (DGEBA) epoxy. The sol-gel technique enables the silica to be successfully incorporated into the network of the hybrids, increasing the thermal stability and improving the mechanical properties of the prepared epoxy/silica nano-hybrids. An autocatalytic mechanism of the epoxy/SiO_2_ nanocomposites was observed. The low reaction rate of epoxy in the nanocomposites is caused by the steric hindrance in the network of hybrids that arises from the consuming of epoxide group in the network of hybrids by the silica. In the nanocomposites, the nano-scale silica particles had an average size of approximately 35 nm, and the particles were well dispersed in the epoxy matrix, according to the TEM images.

## 1. Introduction

Epoxies have many favorable properties such as high tensile strength and modulus, excellent chemical resistance, and high thermal stability. These characteristics make them ideal matrices for many applications, such as epoxy molding compounds (EMCs) [1], printed circuit boards (PCBs) [2], adhesives [3], paints [4], and high-performance composites [5]. Epoxy composites, which are hybrid organic-inorganic materials, have a wide range of engineering applications that require a high strength to weight ratio, low cost and ease of fabrication [6,7].

Sol gel-derived products have various applications, such as thin films [8,9], protective coatings [8], decorative coatings [10], electro-optic components [11] and composites [12]. Sol-gel methods are used to fabricate metal oxide from a chemical solution that acts as the precursor of an integrated network of either discrete particles or network polymers. Typical precursors are metal alkoxides and metal chlorides, which undergo various hydrolysis and polycondensation reactions [13,14]. An extensively studied alkoxide is tetraethyl orthosilicate (TEOS). Alkoxides are ideal chemical precursors for sol-gel synthesis because they react readily with water. The mechanism of the sol-gel process is as follows.

Hydrolysis:

$$\mathrm{Si}{\left(\mathrm{OR}\right)}_{4}+\;H_{2}O\to \mathrm{HO}–\mathrm{Si}{\left(\mathrm{OR}\right)}_{3}+\;R–\mathrm{OH}$$

Condensation:

$${\left(\mathrm{OR}\right)}_{3}–\mathrm{Si}–\mathrm{OH}+\mathrm{HO}–\mathrm{Si}–{\left(\mathrm{OR}\right)}_{3}\to \left[{\left(\mathrm{OR}\right)}_{3}\mathrm{Si}–O–\mathrm{Si}{\left(\mathrm{OR}\right)}_{3}\right]\;+\;H–O–H$$

Polymerization is associated with the formation of a one-, two- or three-dimensional network of siloxane (Si–O–Si) bonds, accompanied by the formation of H–O–H and R–O–H species [15,16,17,18,19].

Epoxy/silica hybrids that comprise nanoparticles that are dispersed throughout an epoxy matrix have been widely studied because they exhibit a desired combination of the flexibility of epoxy and the hardness of silica nanoparticles [20,21]. The sol-gel process is an efficient means of preparing metal oxide networks at low temperatures. The structure of the inorganic material can be controlled by optimizing the synthetic conditions, including concentration, solvent/alkoxide ratio, temperature, pH, and the species of the catalyst and the solvent [22,23]. Whereas the curing kinetics and mechanisms of reaction of various epoxy resins have been studied using various methods, including infrared spectroscopy and differential scanning calorimetry. In fact, differential scanning calorimetry is more commonly used. The effects of nano-scale silica in epoxy resins on their curing kinetics have rarely been investigated. This study provides such critical information to elucidate the reactivity and to predict the curing behavior of epoxy/silica hybrids.

## 2. Experimental Section

### 2.1. Materials and Methods

The epoxy resin that is used in this work is the diglycidyl ether of bisphenol A (DGEBA) (shell 828, Shell Inc., Kawasaki, Japan). The epoxy resin was cured with 2.4-methylhexanhydrophthalic anhydride (MHHPA) (Shell Inc., Kawasaki, Japan). The alkoxides, 4.3-isocyanatopropyltriethoxysilane (IPTES) and tetraethoxysilane (TEOS), were purchased from Shin-Etsu Inc. (Naoetsu, Japan). The chemical structures of DGEBA, MHHPA, IPTES and TEOS are as follows.


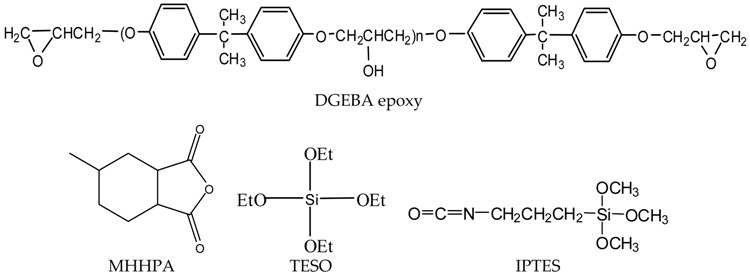


**Scheme 1:**
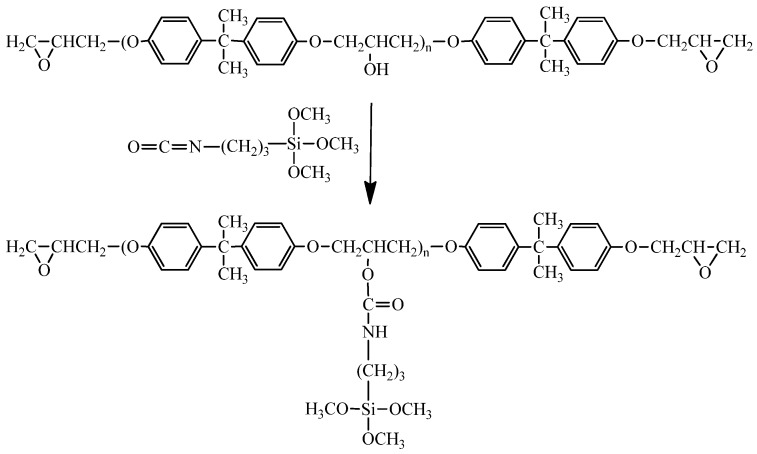
Preparation of silanized epoxy precursor.

**Scheme 2:**
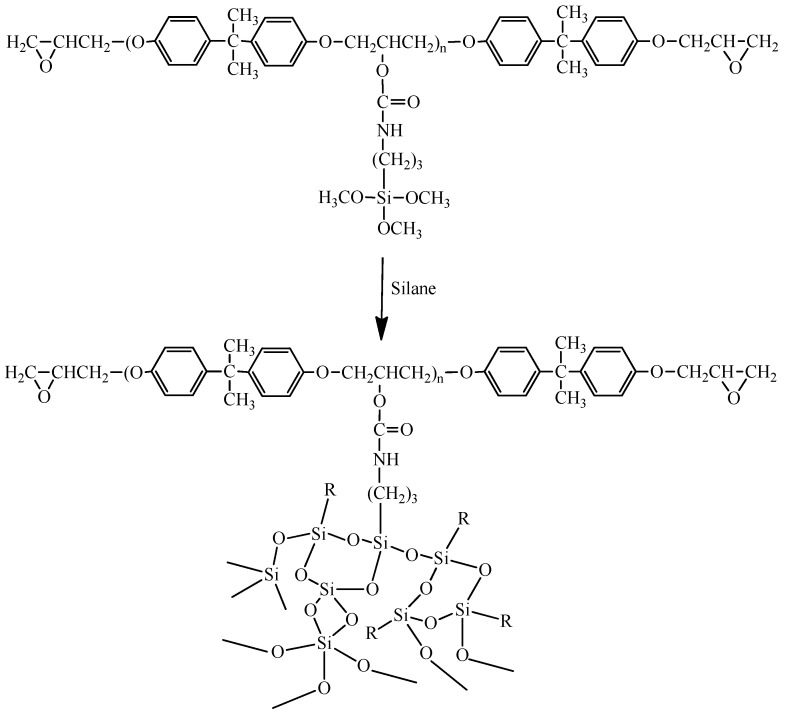
Preparation of epoxy/silica nano-hybrids.

A mixture of epoxy and IPTES, with an epoxy/IPTES weight ratio of 100/5, was stirred vigorously at 80 °C for 2 h to yield the silanized epoxy. Scheme 1 shows the preparation of silanized epoxy precursor. The silanized epoxy, TEOS, water, ethanol, and catalyst were mixed and stirred vigorously at room temperature for 1 h to obtain a homogeneous mixture. The amount of MHHPA that was required to provide an overall –COOH/epoxide molar ratio of 1/1 was then added to the mixture at room temperature and mixing was continued for another 5 min. The curing process was carried out at various temperatures to obtain cured poxy/SiO_2_ hybrids. Scheme 2 shows the process for preparation of epoxy/silica nano-hybrids.

### 2.2. Characterization of Epoxy/Silica Nano-Hybrids

A differential scanning calorimeter (Perkin-Elmer PYRIS I, equipped with an intracooler) was used in the isothermal curing experiments to obtain data for analysis. The isothermal curing reaction was conducted at five temperatures (100 °C, 110 °C, 120 °C, 130 °C, and 140 °C); it was considered to be complete when the isothermal DSC thermogram leveled off to the baseline. The total area under the exothermal curve, which was based on the extrapolated baseline at the end of the reaction, was used to calculate the isothermal heat of curing, Δ*H*_IO_ (Jg^−1^). To determine the residual heat of reaction, Δ*H*_R_ (Jg^−1^), the cured samples were scanned at 10 °C/min from 40 °C to 200 °C. The sum of both the isothermal heat (Δ*H*_IO_) and the residual heat (Δ*H*_R_) of reaction was taken to represent the total heat of reaction (Δ*H*_T_). The isothermal conversion at time *t* was defined as α_I_(*t*) = Δ*H*_I_(*t*)/Δ*H*_T_.

Solid-state ^29^Si NMR measurements were made at room temperature using a Bruker Avance 400 NMR spectrometer that was operated at 400 MHz. The chemically modified silica was examined using solid-state ^29^Si cross-polarization and magic angle spinning nuclear magnetic resonance spectroscopy. The morphology of epoxy/silica nanocomposites was analyzed by a transmission electron microscopy (TEM, model JEOL JEM 1200 EX, Tokyo, Japan) with an accelerating voltage of 100 kV.

## 3. Results and Discussion

### 3.1. Characterization of Epoxy/Silica Nano-Hybrids

**Figure 1:**
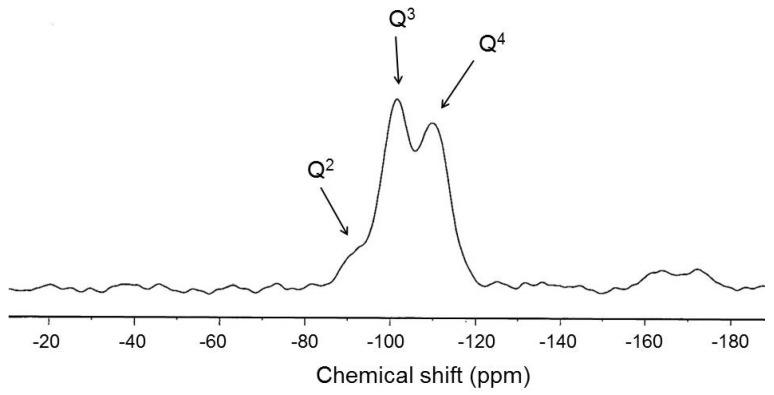
The solid-state ^29^Si NMR spectra of epoxy/silica nanocomposite.

**Scheme 3:**
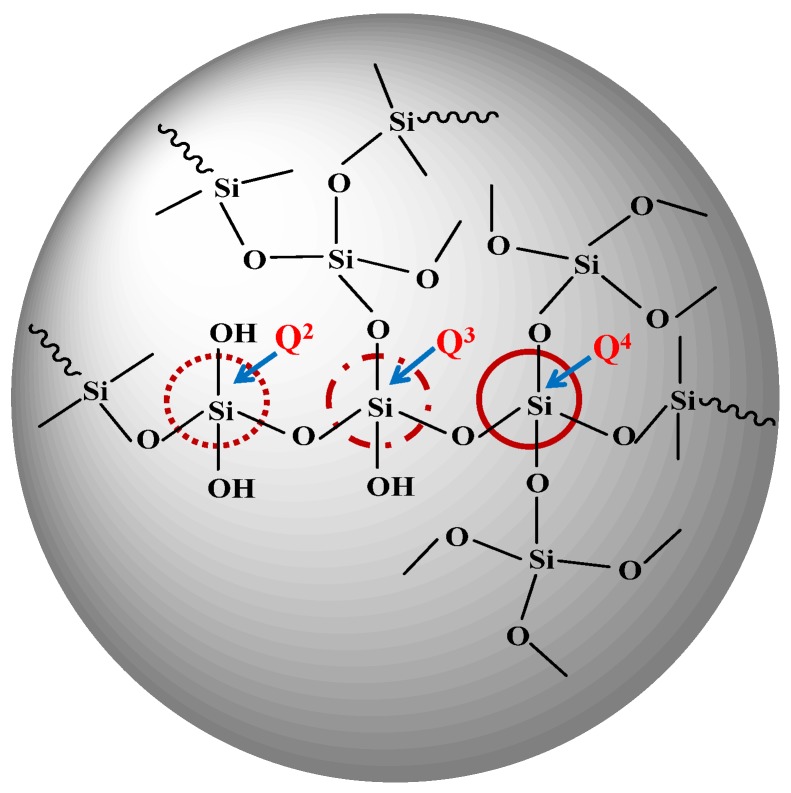
The Q structure of polysilsesquioxane.

The chemical structure of silica in the epoxy/silica nanocomposites was examined using ^29^Si NMR. Figure 1 shows the solid-state ^29^Si NMR spectra of the epoxy/silica nanocomposite, which exhibited major chemical shifts at −91 ppm, −102 ppm, and −110 ppm, which were attributed to silsesquioxane absorption by dihydroxy-substituted silica (Q^2^), monohydroxy-substituted silica (Q^3^) and nonhydroxy-substituted silica (Q^4^), respectively. These chemical shifts involved the Si–O–Si bonding of the silsesquioxane [24,25]. Scheme 3 shows the Q architecture of the polysilsesquioxane in the epoxy/silica nano-hybrids. Siloxane bridges in the silica, which were highly crosslinked in the epoxy/silica hybrid materials, were observed. Since the samples, having a Q^3^ and Q^4^ environment could be considered to include a highly condensed silica phase, the formation of the SiO_2_ network structure was complete in the prepared samples. The incomplete reaction of siloxane was not observed in the epoxy/silica nano-hybrids. Additionally, the amount of Q^4^ was observed to increase with TEOS content, indicating that a large amount of introduced TEOS may have promoted the formation of the silica network.

### 3.2. Cure Kinetics of Epoxy/Silica Nano-Hybrids

A general equation for the autocatalytic curing reactions of many epoxy systems is as follows [7].

(1) $$r=d\alpha /dt=(k_{1}+k_{2}\alpha^{m}){\left(1-\alpha \right)}^{n}$$

where α is the conversion; *k*_1_ and *k*_2_ are the apparent rate constants; *r* is the rate of the reaction, and *m* and *n* are the kinetic exponents of the reactions. The constant *k*_1_ in Equation (1) can be calculated if the initial reaction rate at α = 0 can be estimated. Kinetic constants *k*_1_ and *k*_2_ are assumed to be of the Arrhenius form: $k_{1}=A_{1}EXP(-E_{a1}/RT)$ and $k_{2}=A_{2}EXP(-E_{a2}/RT)$, where A is the pre-exponential constant; *E_a_* is the activation energy; *R* is the gas constant, and *T* is the absolute temperature.

**Figure 2:**
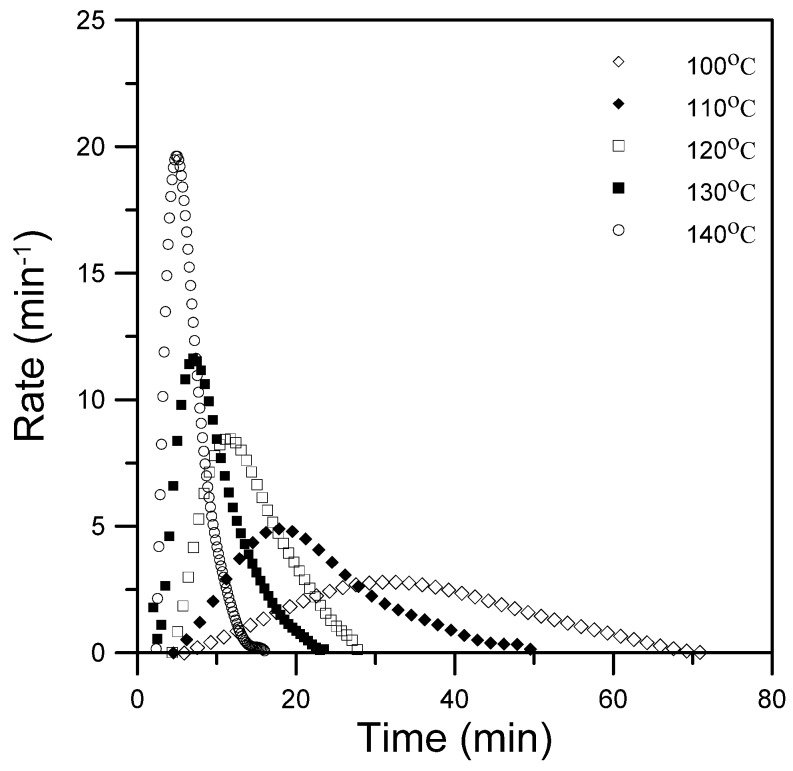
Plot of the reaction rate *vs.* time for epoxy/silica hybrids at five various temperatures.

The epoxy resin contained 10 phr (based on 100 parts of the epoxy) of silica and was cured at five isothermal temperatures of 100 °C, 110 °C, 120 °C, 130 °C, and 140 °C. Kinetic analysis was performed using the above kinetic models. Figure 2 plots the rate curves that were obtained at the five isothermal temperatures. These rate curves were distinctly autocatalytic, and the maximum rates were reached 5 min, 7 min, 11 min, 18 min, and 31 min after the start of the reaction at isothermal reaction temperatures of 100 °C, 110 °C, 120 °C, 130 °C, and 140 °C, respectively. The results in these figures also indicate that the presence of silica in the epoxy does not change its autocatalytic nature.

**Figure 3:**
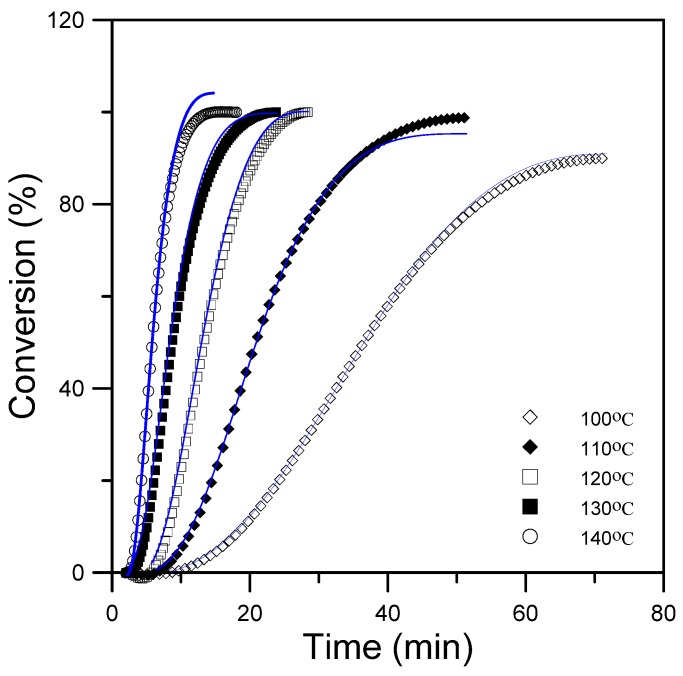
Plot of the reaction rate *vs.* time for epoxy/silica hybrids at five various temperatures.

Table 1 presents the rate constants, which were obtained by iterative and graphic procedures. The orders of the reaction, m and n, were approximately 0.5 and 1.4, respectively, and their values did not vary much among the epoxy-silica hybrids. The value Δ*E*_1_ and Δ*E*_2_ for anhydride-cured DGEBA epoxy that were obtained in this study were 80 kJmol^−1^ and 60 kJmol^−1^, respectively. The autocatalytic kinetic model and the obtained rate constants were used to calculate the empirical curves of conversions as a function of time for the epoxy/silica hybrids at five isothermal curing temperatures. Figure 3 shows that the empirical conversion curves fit the experimental data quite closely until the curing reactions had progressed to the point of vitrification. The model apparently describes the kinetics accurately, but diffusion control in the vitrified state limited the extent of the epoxy reactions.

**Figure 4:**
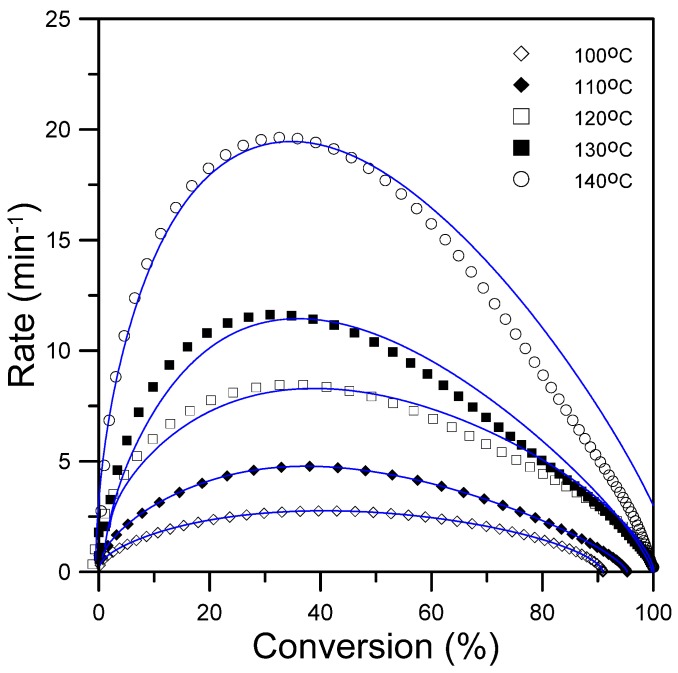
Plot of the reaction rate *vs.* conversion for epoxy/silica hybrids at five various temperatures.

Figure 4 plots the curves of rate *vs.* conversion for the epoxy-silica hybrids with 10 phr silica, which were cured at five isothermal temperatures. The conversion rate above which the curing rate varied coincided with the formation of the gel point, at which the curing reaction of the gelled epoxy network began to slow down.

**Table 1:** Autocatalytic model constants for epoxy–silica hybrids.

*T* (°C)	*m*	*n*	*k*_1_ (min^−1^)	*k*_2_ (min^−1^)	*E_a_*_1_ (kJmol^−1^)	*E_a_*_2_ (kJmol^−1^)	A_1_	A_2_
100	0.5	0.9	0.3	6.4	–	–	–	–
110	0.6	1.0	0.6	14.0	–	–	–	–
120	0.6	1.0	1.2	18.8	5.6	5.4	287	4699
130	0.6	1.0	1.6	30.0	–	–	–	–
140	0.6	0.9	1.9	45.2	–	–	–	–

### 3.3. Morphological Analysis

**Figure 5:**
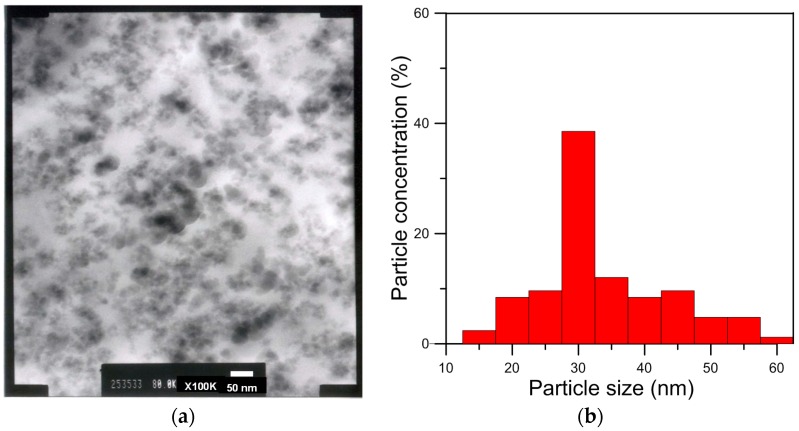
Transmission electron micrograph (TEM) of epoxy/silica hybrids with 10 phr silica: (**a**) TEM image and (**b**) the particle sizes distribution of silica.

**Figure 6:**
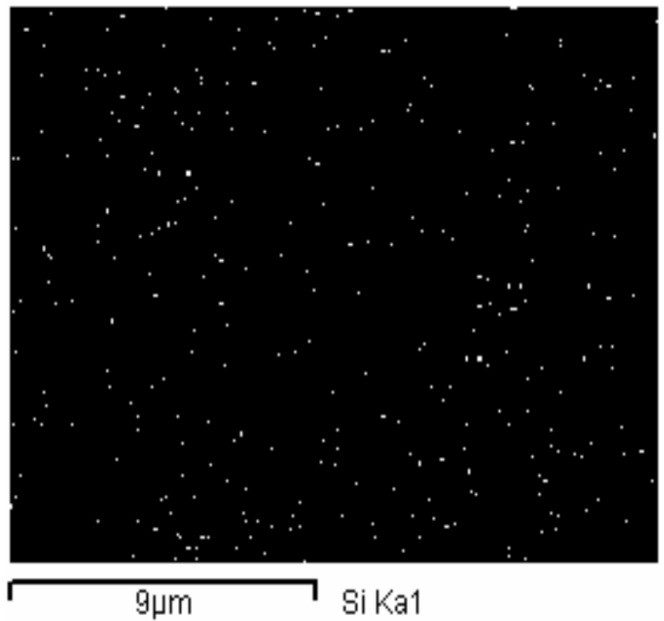
The silicon mapping of epoxy/silica hybrids with 10 phr silica.

Figure 5 shows a TEM image of the silica/epoxy hybrid with 10 phr silica and the histogram of the particle sizes of silica that was cured at 140 °C for 60 min. TEM data reveal that the sizes of the nano-scale silica particles that were synthesized by the sol-gel method varied in the range 15–60 nm and the average size was 35 nm. In the epoxy/silica hybrid, nano-scale silica particles were dispersed uniformly throughout the epoxy matrix. This morphology reveals that 10% TEOS sufficed to form silica particles during the gel reaction. However, the amount of silica particles formed increased with the amount of charged TEOS. The sol-gel reaction changed the sizes of the nano-silica particles. The reactions involved various degrees of hydrolysis and condensation, yielding nano polysilsesquioxane particles of various sizes. Alkoxide addition to epoxy resins is thus expected to change not only their morphology but also the curing kinetics. Phase separation occurs and a heterogeneous morphology develops if the silica and the crosslinked epoxy are not miscible after curing. Additionally, phase morphology development during curing is expected to influence significantly the properties of the cured epoxy resins. Figure 6 show the silicon mapping of silica/epoxy hybrids with 10 phr silica. The silicon distribution mapping of the epoxy/silica hybrids revealed well mixed silicon, suggesting that the inorganic silica nano-particles in the epoxy/silica nanocomposites were distributed uniformly on the nanometer scale and their nano-architectures were well defined.

## 4. Conclusions

A new class of epoxy/silica nano-hybrids was prepared using a sol-gel technique. To improve the compatibility between the organic and inorganic phases, a coupling agent was used to modify the diglycidyl ether of bisphenol A (DGEBA) epoxy. Siloxane bridges in the silica, which were highly crosslinked in the epoxy/silica hybrid materials, were observed. The formation of the SiO_2_ network structure in the prepared samples was complete. The difference in polarity between the inorganic and organic materials may have formed the nanophase-separated structure, which is shown in the TEM microphotographs. The sizes of the nano-scale silica particles in the nanocomposites varied in the range of 15–60 nm, averaging 35 nm, and the particles were well dispersed in the epoxy matrix, according to the TEM images. An autocatalytic mechanism of the curing of 10 phr epoxy/silica hybrids at various isothermal temperatures was observed. Kinetic parameters for the epoxy/silica hybrids were obtained and the proposed kinetic model was found to describe accurately the curing behavior of the epoxy/silica hybrids up to the vitrification point. A kinetic modeling approach is used to demonstrate how thermosetting epoxy resins that have been modified with silica behave during curing. The curing kinetics were corrected for the morphological development of the heterogeneous epoxy/silica hybrids during the curing progress.
